# Identification of genes encoding ALMT and MATE transporters as candidate aluminum tolerance genes from a typical acid soil plant, *Psychotria rubra* (Rubiaceae)

**DOI:** 10.7717/peerj.7739

**Published:** 2019-09-25

**Authors:** Akira Iguchi, Kazutsuka Sanmiya, Kenta Watanabe

**Affiliations:** 1 Geological Survey of Japan, National Institute of Advanced Industrial Science and Technology, Tsukuba, Ibaraki, Japan; 2 Department of Bioresources Engineering, National Institute of Technology, Okinawa College, Nago, Okinawa, Japan; 3 Science and Technology Division, National Institute of Technology, Okinawa College, Nago, Okinawa, Japan

**Keywords:** *Psychotria*, Plant, Acid soils, Transcriptome, MATE, ALMT

## Abstract

To understand how tropical plants have adapted to acid soils, we analyzed the transcriptome of seedlings of* Psychotria rubra*, a typical species found on acid soils. Using RNA-seq, we identified 22,798 genes, including several encoding proteins of the Al^3+^-activated malate transporter (ALMT) and multidrug and toxic compound extrusion (MATE) families. Molecular phylogenetic analysis of ALMTs and MATEs revealed the grouping of those from *P. rubra*, which may be useful to select targets for elucidating the molecular basis of *P. rubra* adaptation to acid soils in the future. The transcriptome datasets obtained in this study would help us to further understand the physiological and ecological aspects of soil adaptation of *Psychotria* species.

## Introduction

Understanding how plants adapt to various soils is essential in plant biology (Hiradate, Ma & Matsumoto, 2007) because plants are sessile and need to grow roots in settled soils. Adaptation to acid soils is an important issue because acid soils cover a considerable part of Earth’s arable land and prevent agriculture of most plants (Von Uexküll & Mutert, 1995). In acid soils, aluminum is toxic to root tip growth, and various aspects, from molecular to physiological, of the mechanisms of aluminum toxicity have been explored in detail (reviewed in Ma, 2007).

Proteins of the Al^3+^-activated malate transporter (ALMT) and multidrug and toxic compound extrusion (MATE) families are likely involved in plant adaptation to acid soils (Delhaize, Gruber & Ryan, 2007; Delhaize, Ma & Ryan, 2012; Ma, 2007; Ma, Chen & Shen, 2014). ALMTs and MATEs release organic acids (malate and citrate, respectively), which bind Al^3+^ and detoxify it. ALMTs and MATEs related to aluminum tolerance have been identified in model and agricultural plants (wheat: Sasaki et al., 2004; barley: Delhaize et al., 2004; Furukawa et al., 2007; maize: Maron et al., 2010; *Arabidopsis*: Hoekenga et al., 2006; Liu et al., 2009), but the composition of those families in non-model wild plants has hardly been explored.

*Psychotria* (Rubiaceae) is a highly diversified genus comprising more than 1,600 species distributed in all tropical and some subtropical regions (Hamilton, 1989; Davis et al., 2001; Razafimandimbison et al., 2014). Because *Psychotria* species adapt to several types of soils (e.g., soils with high concentrations of nickel; Merlot et al., 2014), the genus is an ideal target to use to understand how adaptation of wild plants to different types of soils has evolved. In this study, we report ALMTs and MATEs of *P. rubra*, which grows on acid soils (Miyawaki, 1989).

## Materials and Methods

### Sampling, RNA extraction, RNA-seq library preparation, and sequencing

**Figure 1:**
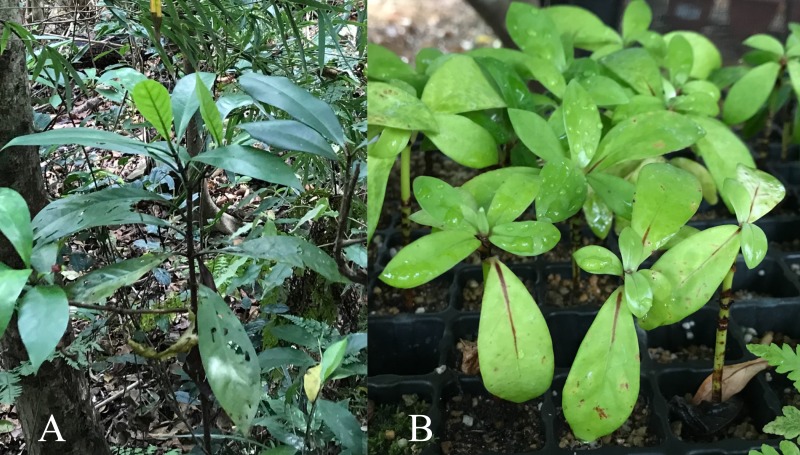
*Psychotria rubra*. (A) Shrub on Mount Nago-dake, Okinawa, Japan. (B) Seedlings.

Seeds of *P. rubra* were collected on Mt. Nago-dake, in the north of Okinawa Island, and seedlings were grown in a greenhouse of the National Institute of Technology, Okinawa College (Fig. 1). RNA was extracted from the seedlings (three–five cm height) using an RNeasy Plant Mini kit (Qiagen, Hilden, Germany). An RNA-seq library was prepared using a TruSeq Stranded mRNA Sample Prep Kit (Illumina, San Diego, CA, USA). The library was sequenced (100-bp paired-end reads) on an Illumina HiSeq 2500 platform. The above procedures on RNA-seq were outsourced to Hokkaido System Science Corporation, Japan.

### De novo assembly and annotation of transcriptome sequences

FASTQ files were filtered, the reads with poor-quality bases (*Q* < 20) and those shorter than 20-bp were excluded, and adapter sequences were removed in cutadapt v. 1.9.1 software (Martin, 2011). PCR duplicates that arose during library preparation were removed by ConDeTri software (Smeds & Künstner, 2011). The remaining paired-end reads were assembled in Trinity v. 2.8.4 software (Grabherr et al., 2011) with the default options. Open reading frames of >150 amino acids were identified by using TransDecoder software (Haas et al., 2013) and the results of BLASTP searches within the Swiss-Prot database (*e*-value <1e^−5^). Redundant amino acid sequences were removed in CD-HIT v. 4.7 software (−c 0.95; Li & Godzik, 2006). The remaining sequences were used as queries in BLASTP searches (*e*-value <1e^−5^) of *Arabidopsis thaliana* sequences (TAIR10; Swarbreck et al., 2007) and selected sequences assumed to be *P. rubra* itself. Basic information on sequences was obtained in SeqKit v. 0.9.3 software (Shen et al., 2016). Sequence data are accessible under the DNA Data Bank of Japan (DDBJ) Sequence Read Archive (accession: DRA008339). The raw data has been also available at Figshare (https://doi.org/10.6084/m9.figshare.7848425.v2).

**Table 1:** Summary of de novo assembly.

Number of contigs	131,578
Total bases (bp)	161,583,381
Longest contig length (bp)	16,195
Shortest contig length (bp)	185
Average contig length (bp)	1,228
N50	2,007

**Table 2:** Summary of blastp of TaALMT1 and HvAACT1 against *P. rubra* amino acid sequences and annotation of *P. rubra* sequences from the Swiss-Prot database.

Gene name	Sequence ID	% identity	*e*-value	Bit score	Annotate description (Swiss-prot)	Accession no. (Swiss-prot)	*e*-value (Swiss-prot)
TaALMT1	Prub_08163	45.966	1.05E−110	334	Aluminum-activated malate transporter 2 (AtALMT2)	Q9SJE8	3.67E−166
TaALMT1	Prub_08164	49.477	4.78E−90	277	Aluminum-activated malate transporter 2 (AtALMT2)	Q9SJE8	1.12E−133
TaALMT1	Prub_10125	43.902	1.19E−77	251	Aluminum-activated malate transporter 12 (AtALMT12) (Quick anion channel 1)	O49696	0
TaALMT1	Prub_08553	36.111	3.39E−63	214	Aluminum-activated malate transporter 9 (AtALMT9)	Q9LS46	0
TaALMT1	Prub_16333	33.333	6.06E−57	197	Aluminum-activated malate transporter 9 (AtALMT9)	Q9LS46	0
TaALMT1	Prub_03305	51.19	5.72E−53	176	Aluminum-activated malate transporter 10 (AtALMT10)	O23086	3.21E−59
TaALMT1	Prub_02168	33.772	4.74E−23	98.6	Putative aluminum-activated malate transporter 3 (AtALMT3)	Q9LPQ8	1.74E−74
TaALMT1	Prub_02171	27.801	1.10E−22	98.6	Aluminum-activated malate transporter 9 (AtALMT9)	Q9LS46	1.99E−141
TaALMT1	Prub_16334	39.655	1.03E−19	86.3	Aluminum-activated malate transporter 9 (AtALMT9)	Q9LS46	6.62E−51
TaALMT1	Prub_08405	30.108	2.45E−19	88.6	Aluminum-activated malate transporter 9 (AtALMT9)	Q9LS46	9.01E−62
TaALMT1	Prub_08554	51.852	6.42E−12	63.2	Aluminum-activated malate transporter 9 (AtALMT9)	Q9LS46	3.20E−27
TaALMT1	Prub_02170	39.062	4.77E−11	62	Aluminum-activated malate transporter 9 (AtALMT9)	Q9LS46	7.28E−32
TaALMT1	Prub_02169	30	2.57E−07	51.6	Aluminum-activated malate transporter 4 (AtALMT4)	Q9C6L8	1.57E−73
TaALMT1	Prub_02167	38.129	3.26E−07	50.8	Aluminum-activated malate transporter 9 (AtALMT9)	Q9LS46	3.96E−26
HvAACT1	Prub_03062	57.752	0	537	Protein DETOXIFICATION 42 (AtDTX42) (Aluminum-activated citrate transporter) (AtMATE) (FRD-like protein) (Multidrug and toxic compound extrusion protein 42) (MATE protein 42)	Q9SYD6	0
HvAACT1	Prub_17943	55.955	0	536	Protein DETOXIFICATION 42 (AtDTX42) (Aluminum-activated citrate transporter) (AtMATE) (FRD-like protein) (Multidrug and toxic compound extrusion protein 42) (MATE protein 42)	Q9SYD6	0
HvAACT1	Prub_17944	59.432	0	531	Protein DETOXIFICATION 42 (AtDTX42) (Aluminum-activated citrate transporter) (AtMATE) (FRD-like protein) (Multidrug and toxic compound extrusion protein 42) (MATE protein 42)	Q9SYD6	0
HvAACT1	Prub_17945	55.894	3.61E−168	485	Protein DETOXIFICATION 42 (AtDTX42) (Aluminum-activated citrate transporter) (AtMATE) (FRD-like protein) (Multidrug and toxic compound extrusion protein 42) (MATE protein 42)	Q9SYD6	0
HvAACT1	Prub_03064	63.372	4.58E−147	429	Protein DETOXIFICATION 43 (AtDTX43) (Multidrug and toxic compound extrusion protein 43) (MATE protein 43) (Protein FERRIC REDUCTASE DEFECTIVE 3) (AtFRD3) (Protein MANGANESE ACCUMULATOR 1)	Q9SFB0	1.12E−160
HvAACT1	Prub_03541	41.322	1.43E−120	368	Protein DETOXIFICATION 45, chloroplastic (AtDTX45) (Multidrug and toxic compound extrusion protein 45) (MATE protein 45)	Q9SVE7	0
HvAACT1	Prub_18165	39.506	2.75E−114	350	Protein DETOXIFICATION 45, chloroplastic (AtDTX45) (Multidrug and toxic compound extrusion protein 45) (MATE protein 45)	Q9SVE7	0
HvAACT1	Prub_03065	54.4	6.52E−55	186	Protein DETOXIFICATION 42 (AtDTX42) (Aluminum-activated citrate transporter) (AtMATE) (FRD-like protein) (Multidrug and toxic compound extrusion protein 42) (MATE protein 42)	Q9SYD6	3.65E−69
HvAACT1	Prub_03063	53.441	4.31E−52	179	Protein DETOXIFICATION 42 (AtDTX42) (Aluminum-activated citrate transporter) (AtMATE) (FRD-like protein) (Multidrug and toxic compound extrusion protein 42) (MATE protein 42)	Q9SYD6	1.00E−66
HvAACT1	Prub_14684	43.541	4.14E−37	137	Protein DETOXIFICATION 44, chloroplastic (AtDTX44) (Multidrug and toxic compound extrusion protein 44) (MATE protein 44)	Q84K71	4.41E−94
HvAACT1	Prub_14685	38.174	2.44E−22	96.7	Protein DETOXIFICATION 44, chloroplastic (AtDTX44) (Multidrug and toxic compound extrusion protein 44) (MATE protein 44)	Q84K71	4.59E−53
HvAACT1	Prub_18629	23.25	7.67E−14	73.6	Protein DETOXIFICATION 46, chloroplastic (AtDTX46) (Multidrug and toxic compound extrusion protein 46) (MATE protein 46) (Protein EDS5 HOMOLOGUE)	Q8W4G3	0

### Extraction of ALMTs and MATEs of *P. rubra* and molecular phylogenetic analysis

We searched for *P. rubra* ALMTs and MATEs by BLASTP (*e*-value <1e^−5^) using sequences of TaALMT1 (UniProt database ID: Q76LB1) and HvAACT1 (UniProt ID: A7M6U2; this was the first MATE identified in barley (Furukawa et al., 2007) as queries). We then performed BLASTP searches against the Swiss-Prot database (*e*-value <1e^−5^) with each ALMT and MATE of *P. rubra* and selected the top 10 hits for each. The amino acid sequences of ALMTs and MATEs from *P. rubra* and related sequences from Swiss-Prot and other studies (Dreyer et al., 2012; Liu et al., 2016) were aligned in MAFFT v. 7.407 software (Katoh & Standley, 2013). We excluded four *P. rubra* ALMT sequences (Prub_02169, Prub_02171, Prub_08405, Prub_08554) from the following analysis because of poor alignment. We selected only plant MATEs, including HvAACT1 and those from (Liu et al., 2016), for the following analysis. Neighbor-joining trees of ALMTs and MATEs were constructed in MEGA7 software (Kumar, Stecher & Tamura, 2016) with the following settings: Poisson model, Uniform rates, and Pairwise deletion. To evaluate the confidence of phylogenetic trees, bootstrap tests were performed with 1,000 replicates.

## Results and Discussion

Our RNA-seq analysis of *P. rubra* yielded 57,110,261 paired-end reads, of which 53,994,410 remained after filtering. De novo assembly of the remaining reads resulted in 131,578 contigs (Table 1), in which we found 24,687 non-redundant amino acid sequences; 22,798 of them were expected to originate from *P. rubra* itself as indicated by BLASTP analysis of TAIR10 (the remaining 1,889 sequences were almost no-hit in Swiss-Prot database or included those from microorganisms, etc). Among these 22,798 sequences, 19,701 ones were hit against Swiss-Prot database, and gene ontology (GO) numbers were found in 1,0348 ones. From these sequences, we found 14 ALMTs and 12 MATEs (Table 2).

**Figure 2:**
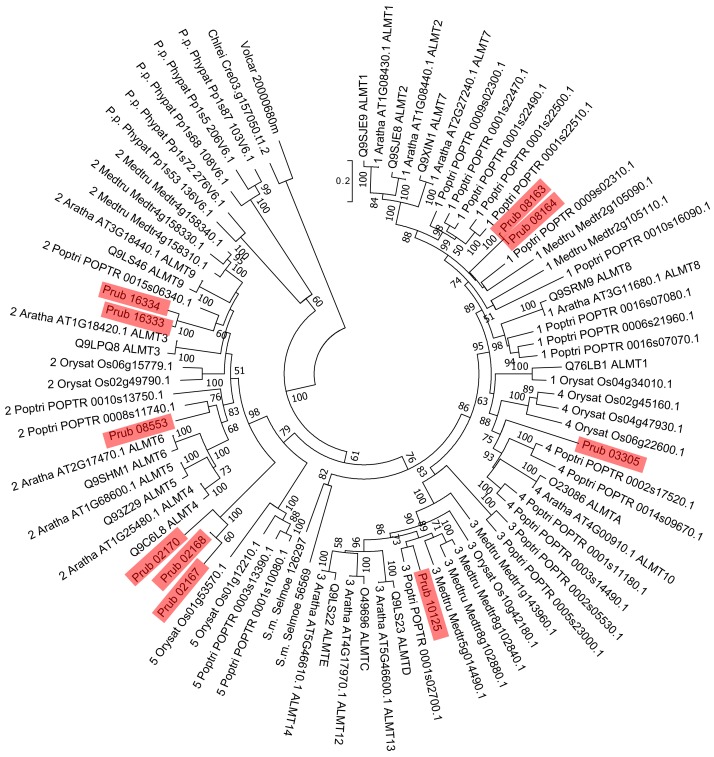
Phylogenetic tree of ALMTs of *P. rubra* and related proteins. Sequences of *P. rubra* are shown using red shades. For sequences other than *P. rubra* ALMTs, the labels show the UniProt database ID and ALMT type, and ones from Dreyer et al. (2012). Number at each node is the bootstrap value.

**Figure 3:**
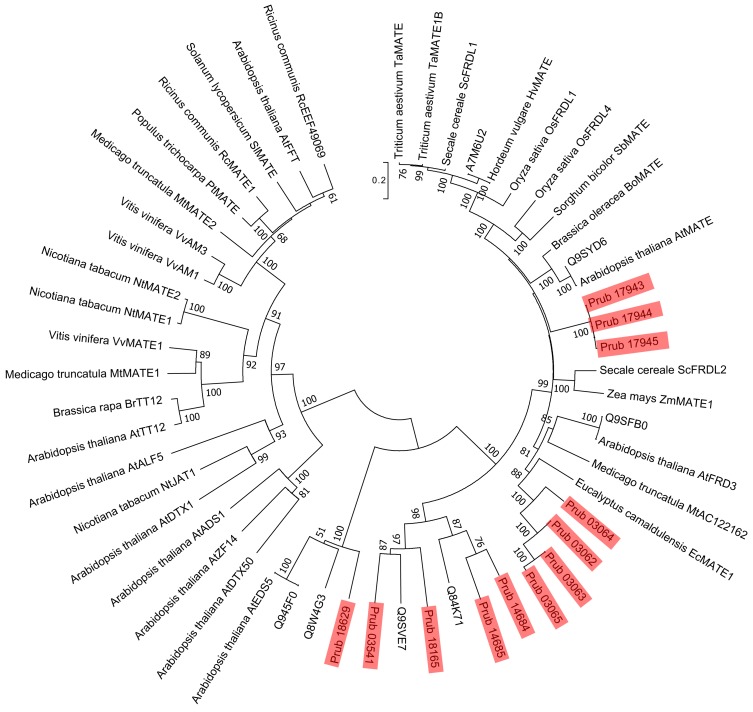
Phylogenetic tree of MATEs of *P. rubra* and related proteins. Sequences of *P. rubra* are shown using red shades. For sequences other than *P. rubra* MATEs, the labels are UniProt database IDs, and ones from Liu et al. (2016). Number at each node is the bootstrap value.

Using 14 ALMTs from *P. rubra* as queries in a BLASTP search against the Swiss-Prot database, we found 14 homologs, all of plant origin (mainly from *Arabidopsis*). Using a similar approach, we found 13 homologs of MATEs (six from *Arabidopsis* and seven from non-plant organisms). Molecular phylogenetic analysis did not detect *P. rubra* orthologs of TaALMT1 (UniProt ID: Q76LB1) (Fig. 2). ScALMT1 (accession number: ABA62397) from rye (*Secale cereale*) is the only known clear ortholog of TaALMT1 (Delhaize, Gruber & Ryan, 2007). ALMT1 of *Arabidopsis* (UniProt ID: Q9SJE9), encoded by an aluminum tolerance gene (Hoekenga et al., 2006), is clearly distinct from TaALMT1 (Delhaize, Gruber & Ryan, 2007). Thus, ALMTs related to aluminum tolerance may have multiple origins. Molecular phylogenetic analysis of MATEs revealed no clear orthologs of HvAACT1 (UniProt ID: A7M6U2) in *P. rubra* (Fig. 3).

Expression and functional analyses of ALMTs and MATEs of *P. rubra* would be useful for understanding their roles in soil adaptation of *Psychotria* (e.g., with and without Al treatment). Another aluminum tolerance mechanism of plants (different from releasing organic acids), aluminum accumulation, has been reported in several species of the Rubiaceae (Jansen et al., 2003). Genes related to this function are also good targets for future studies to explain the molecular basis of acid soil adaptation of *P. rubra*.

## Conclusions

We succeeded in identifying transcriptome sequences including ALMTs and MATEs from *P. rubra* in this study. Comparative transcriptome analysis of several *Psychotria* species would help us to clarify the physiological and ecological aspects of diversification of this genus (e.g., adaptation to metalliferous soils; Merlot et al., 2014). In particular, *Psychotria manillensis*, which is closely related to *P. rubra*, is reportedly adapted to non-acid soils (Miyawaki, 1989). Thus, comparative analysis of *P. rubra* and *P. manillensis* should help to explain how soil adaptation–related genes are involved in adaptive evolution of *Psychotria* species.

## Additional Information and Declarations

### DNA Deposition

The following information was supplied regarding the deposition of DNA sequences: Sequence data are accessible at the DNA Data Bank of Japan (DDBJ) Sequence Read Archive: DRA008339.

### Data Availability

The following information was supplied regarding data availability: The raw data is available at Figshare: Iguchi, Akira (2019): Transcriptome of *Psychotria rubra* (Rubiaceae). figshare. Dataset. https://doi.org/10.6084/m9.figshare.7848425.v2.
